# Registration and Fusion of the Autofluorescent and Infrared Retinal Images

**DOI:** 10.1155/2008/513478

**Published:** 2008-10-21

**Authors:** Radim Kolar, Libor Kubecka, Jiri Jan

**Affiliations:** Department of Biomedical Engineering, Faculty of Electrical Engineering and Communication, Brno University of Technology, Kolejni 4, 61200 Brno, Czech Republic

## Abstract

This article deals with registration and fusion of multimodal opththalmologic images obtained by means of a laser scanning device (Heidelberg retina angiograph). The registration framework has been designed and tested for combination of autofluorescent and infrared images. This process is a necessary step for consecutive pixel level fusion and analysis utilizing information from both modalities. Two fusion methods are presented and compared.

## 1. INTRODUCTION

Glaucoma is the second most frequent cause of permanent blindness in industrial developed countries [1]. It is caused by an irreversible damage of the optical nerve connected with degeneration of retinal ganglia cells, axons (neural fibres), and gliocells (providing nutrition for the axons). If not diagnosed in early stage, the damage of the optical nerve becomes permanent, which in the final stage may lead to blindness. The number of people with open-angle and angle-closure glaucoma in 2020 is estimated to 79.6 million [2]. Development of new diagnostic approaches that would enable early detection of glaucoma development is therefore very desirable. One important view is that these new methods are to be applicable in routine diagnostic process, they should be robust and fast, particularly for screening programs.

During the last years, there is a clear tendency in medicine to combine (fuse) data from different diagnostic sources (e.g., X-ray, CT, PET). Similar situation is also in the area of ophthalmologic diagnostic methods. Besides registration and fusion of standard photos of the eye fundus and data from confocal ophtalmoscope (e.g., [3]), the images obtained under various imaging conditions are investigated, as different modalities. One of the promising approaches that could support early diagnosis of the glaucoma is the use of combined retinal autofluorescence (AF) images and infrared (IR) images. This method utilizes information about hyperfluorescent zones in AF image (which is a symptom of glaucoma in its early stage [4]) and information about position of the optical nerve head from IR images [5].

This fusion application is based on properly registered image data. Obviously, multimodal registration is needed and one has to find a specific registration framework, including also the proper image preprocessing. Such a framework is a trade-off between registration accuracy and computational demands as shown in previous papers [6–8]. In this article, we propose a registration method (Section 3) for the autofluorescent and infrared retinal images (Section 2); its evaluation on an available image set is also presented (Section 4). The direct application of the registered images is presented by means of pixel level image fusion (Section 5).

## 2. IMAGE DATA

The images, for which the algorithms have been designed, were acquired at the Department of Ophthalmology, Friedrich-Alexander University of Erlangen-Nurnberg, Germany. The laser scanning ophthalmoscope used for the acquisition was the Heidelberg retina angiograph2 (HRA2), which serves for angiographic examination of human retina. Several modes of the examination are available, for example, fluorescein angiography, indocyanine green angiography, infrared imaging [9]. All images, provided by either of the modalities, may be denoted as scanning laser ophthalmoscopic (SLO) images.

### 2.1. Autofluorescence images

**Figure 1:**
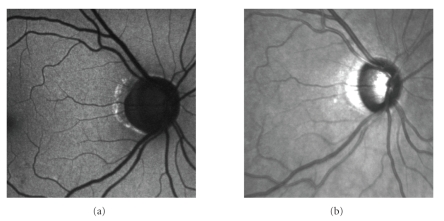
(a) AF and (b) IR images.

In AF mode, the retina is illuminated by a narrow blue light laser beam (*λ* = 488 nm) in a raster manner. This beam excites the lipofuscin that consequently emits light with a longer wavelength (around 500 nm) [10]. The emission intensity depends on the amount of lipofuscin accumulation in retinal pigment epithelium (RPE). There are some studies showing the correlation between the lipofuscin accumulations around the optic disc (OD) and the stage of the optic disc atrophy [11]. An example of an AF image is shown on Figure 1(a). The higher level of autofluorescence is predominantly observed at the border of the atrophic zone alpha, according to the classification used by ophthalmologists.

### 2.2. Infrared images

In IR mode, HRA2 device uses infrared laser light with wavelength *λ* = 820 nm to illuminate the tissue in raster manner. During scanning, the reflections from tissue are acquired. The IR image has the same size and resolution as the AF image. An example of an IR image is shown on Figure 1(b).

Because the AF and IR images are recorded in time sequence, the patient's eye can move, which causes a spatial shift between the images or even inside a gradually scanned image. To be able to fuse the information from both images, a multimodal registration (e.g., [12, 13]) must be performed. This is partly simplified by identical image resolution in both modalities (10 *μ*m/pixel and size 512 × 512 pixels for low resolution mode and 5 *μ*m/pixel, size 1024 × 1024 pixels for high resolution mode).

## 3. IMAGE REGISTRATION

General view of registration methods applied to various types of image data (CT, PET, MRI, etc.) can be found in several review papers [14–17] or books [18, 19]. Specific methods for the retinal image registration are also described in several application papers [12, 13, 20–25].

One of the widely used methods is the registration based on feature correspondence. Zana and Klein [21] describe multimodal method based on segmentation of vessel tree in fluorescent retinal angiography combined with fundus photograph (FP). The bifurcation points of the vessel tree are detected and used as features for the registration process. In [22], Can describes an algorithm for constructing a mosaic consisting of several curved fundus photographs. A 12-parameter spatial transformation is used. Similar method is mentioned in Stewart et al. [13] where authors designed feature correspondence registration for retina mosaicing. Laliberté et al. [20] presented a new method for fusion of FP and fluorescein angiography images after registration also based on bifurcation points. Chanwimaluang et al. [12] used the vascular tree extraction in monomodal registration to create a mosaic image. Matsopoulos et al. [23] extracted bifurcation points only in the reference image and used self-organizing maps for minimizing the difference between gradient pixel intensities to find the parameters of the affine transforms.

There are several problems of this registration approach: determination of the landmarks in case of hyperfluorescence, haemorrhages, or drusens. Other problem is connected with landmark correspondence accented in a case of multimodality images, absence of landmarks for small blood vessels at the image periphery, and in a case of low-quality images (blurred or noisy).

In Wachowiak et al. [24], a method for registration of histological cuts is presented. Robustness of a particle swarm optimization in comparison with evolutionary search is discussed. In Rosin et al. [25], the method concerning multimodal registration of confocal SLO and color FP images is presented. It uses mutual information (MI) metric and pyramidal approach in combination with simulated annealing and gradient descent optimizers. Quality of registration was judged subjectively on the scale: “0-poor,” “1-moderate,” “2-good,” “3-excellent,” and the mean score was evaluated. A similar approach has been used in the present work as well as in the previous paper [7].

Image registration can be treated as the optimization process of finding the parameter vector *α*_0_ of the spatial transformation *T*_*α*_ aligning the content of the images:

(1) $$\alpha_{o}\;=\;\arg\{\underset{\alpha }{\max}\;C(f,\;T_{\alpha }(g))\},$$

where *f* denotes the reference (fixed) image, *g* is the transformed (moving) image, and *C* is an optimization criterion which evaluates the registration quality.

Based on the prior knowledge of image properties, all parts of the registration process should be chosen carefully to ensure registration robustness. The components of the registration framework include the following.

- Preprocessing for example, noise suppression and edge enhancement.
- Criterion metrics: selection among mean squared differences, normalized cross-correlation, gradient difference, and mutual information.
- Spatial transformation: rigid or flexible transformation.
- Optimization strategy: for example, gradient-based or population-based methods.
- Interpolation: needed to match the discretization grids of both images: for example, nearest neighbor, bilinear, radial basis functions, and so forth.

In the following, we will briefly comment on each of them with respect to the concrete problem to be solved.

### 3.1. Preprocessing

**Figure 2:**
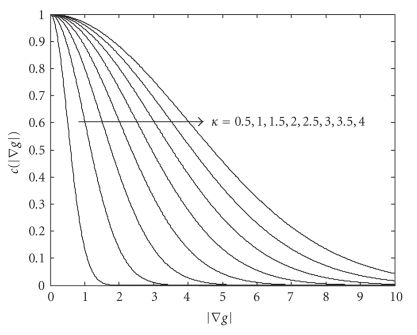
The function shape that affects the behavior of anistropic diffusion in (2).

**Figure 3:**
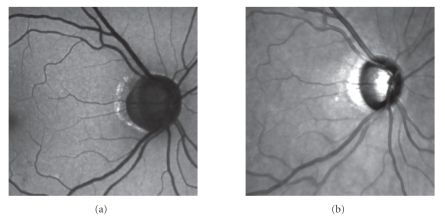
Effect of anisotropic diffusion filtering on (a) AF and (b) IR images. The corresponding images are on Figure 1. The parameters of anisotropic diffusion were set to *κ* = 1.5, *t* = 0.125, and iteration = 10.

The first step is the image preprocessing. Because of the low signal-to-noise ratio (SNR), noise suppression method should be performed, but possibly without blurring the edges which carry information needed for registration. For that reason, we used the anisotropic diffusion introduced by Perona and Malik [26]. The approach of this method is that a Gaussian smoothed image is considered as a single time slice of the solution to the heat equation:

(2) $$\frac{\partial g(x,\;y,\;t)}{\partial t}\;=\;\nabla \cdot c(\left|\nabla g\right|)\nabla g(x,\;y,\;t),$$

where *g*(*x*, *y*, 0) = *f*(*x*, *y*) is the input image. The function *c*(|∇*g*|) is a function that reduces the conductance at the areas of large gradient |∇*g*| . We used the following form [27]:

(3) $$c(\left|\nabla g\right|)\;=\;e^{-|\nabla g{|}^{2}/2\kappa^{2}},$$

which introduces a new conductance parameter *κ*, controlling sensitivity of the smoothing process. The behavior of this function for tested values of *κ* is depicted on Figure 2. The second parameter of anisotropic diffusion is time *t* and the last parameter is the number of iterations. For the IR and AF images, we found as suitable values for *κ* = 1.5, *t* = 0.125, and iteration = 10. For higher values of *κ*, the smoothing effect was too high and the resulting images were not suitable for consequential gradient computation (see Section 3.2). The effect of this filter is shown on Figure 3. 

### 3.2. Similarity metric

The choice of the similarity metric plays a crucial role in the registration process. Because the shift in *x*, *y* axes will be the parameters with the main influence to the value of metrics, we investigated the behavior of different kind of metrics as a function of *x*, *y* shift: the normalized cross-correlation (NCC), the mean of squared differences (MSD), and the mutual information (MI) as defined in [27].

**Figure 4:**
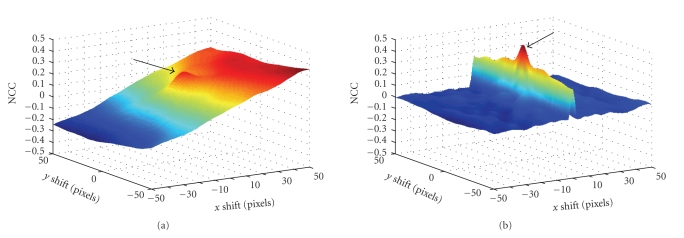
(a) One example of the metric (NCC) surface for original images and (b) corresponding gradient images. The IR and AF images were used and the position of the global maximum is expected position (0,0) pixels, depicted by arrows. Maximum value of the peak on the left image does not correspond to global maximum in contrast to right image.

As expected, the absolute gradient of the filtered image turned out more convenient for registration, using any of the criteria, because it causes more distinct peak of the maximum (see Figure 4 for comparison) and this peak is global in contrast to local maximum on the metric surface obtained for nongradient images. The image gradient computation can be also considered as a preprocessing step. It is a numerical approximation of the gradient absolute value:

(4) |∇F| = (∂F∂xi^)2 + (∂F∂yj^)2.

As can be seen from Figure 4 there is clear visibility of the positive influence to NCC metric in case that the gradient images are used.

**Figure 5:**
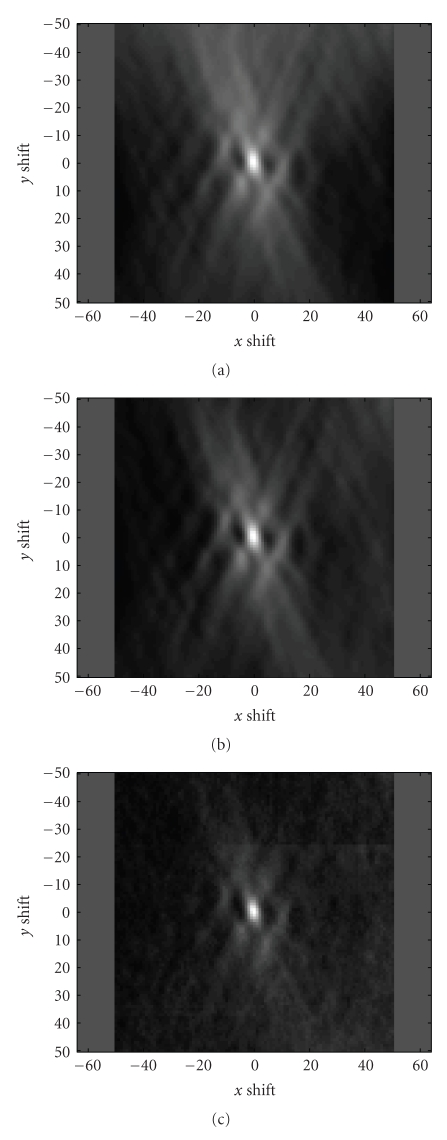
The metric plane shown as 2D images for IR and AF images. The brigthness represents similarity value: (a) MSS, (b) NCC, (c) MI. The proper shift values are located at (0,0) position.

The above-mentioned metrics were evaluated for the defined shifts pixel by pixel, using several pairs of AF and IR images. The examples of these metric surfaces are depicted on Figure 5. One can see that the NCC and MSD similarity metrics have quite similar surfaces. More detailed examination showed that the NCC metric plane has sharper global maximum for majority of the tested images. It also has less local extremes, which is welcome in optimization. Comparing NCC and MI metric planes, the MI metric shows also good properties for optimization. But, considering the computational complexity, we decided to use the NCC metric, according to the definition [27]

(5) $$\text{NCC}(F,\;G)\;=\;\frac{\sum_{i=1}^{N}F_{i}\cdot G_{i}}{\sqrt{\sum_{i=1}^{N}F_{i}^{2}\cdot \sum_{i=1}^{N}G_{i}^{2}}},$$

where *F*_*i*_ and *G*_*i*_ represent the pixel values of gradient images and *N* is number of pixels in the overlapping region. The means were subtracted before NCC computation.

### 3.3. Spatial transformation and image resampling

The spatial transform *T*_*α*_ represents the spatial mapping of pixel values from the moving image space to points in the fixed image space. The type of the transform has to be chosen suitably in order to compensate the distortion between images.

Considering the acquisition process, there can be several types of distortion due to sequential image scanning. One is due to the patient movement between the AF and IR image acquisition, which can be shift and small rotation. The scaling parameters were also included because of the optic of the eye; the slightly different behavior in reflection and refraction for light with different wavelengths. Therefore, we considered only translations in both axes (*t*_*x*_, *t*_*y*_), rotation (*θ*), and scaling (*s*_*x*_, *s*_*y*_) as the transform parameters (see (6)).

Obviously, there can be more complicated discrepancies between images, caused by movement of the eye during the scanning process. These kinds of distortion would need flexible transformations [8]. To make our method practicable (i.e., to speed up the computations), we restrict it to the class of image pairs that are not corrupted during the single-image scanning and thus need affine transform with 5 parameters. The chosen transform can be represented by the transformation matrix for homogeneous coordinates:

(6) $$T_{\alpha }\;=\;\left(\begin{array}{ccc} s_{x}\cdot \cos(\theta ) & -s_{y}\cdot \sin(\theta ) & t_{x}\\ s_{x}\cdot \sin(\theta ) & s_{y}\cdot \cos(\theta ) & t_{y}\\ 0 & 0 & 1 \end{array}\right).$$

The registered image data of the moving image are computed for the coordinates in the target pixels of the fixed image. Therefore, interpolation takes place after each new (possibly optimal) spatial transform is computed. The nearest neighbor, bilinear, and cubic B-splines are the most widely used interpolation functions [14]. The choice of the interpolation technique is more critical in cases where high enlargement of the moving image is employed, which is not our case. We observed that the choice of any of the three interpolation techniques had no visible influence on the registration results. To achieve high computational speed, the nearest neighbor technique was thus used.

### 3.4. Optimization

The registration consists of searching the optimal transform parameters in multidimensional space; in our case, 5-parametric space. In spite of image preprocessing, the surface of similarity metric contains many local extremes. One class of the methods that can cope with this problem is based on the population evolution, where more solutions are considered at one iteration. Each solution has its own similarity metric value. During optimization process (evolution) the samples with a high metric value are replaced with new solutions according to a defined (heuristic) rule. This rule, called “alternation heuristic,” can also change during this process. One of the successful population evolution methods in retina image registration [7] is the controlled random search (CRS) algorithm [28]. The basic version of CRS is shown in Algorithm 1.

It uses a defined heuristic rule *h*(*P*) to generate a new sample in population. If this new point in the search space has a better similarity metric value, it replaces the worst sample in population. The heuristic rule *h*(*P*) can be based on evolutional strategy, simplex reflection, or differential evolution and can also alternate between them during the registration process [28, 29]. The above-mentioned heuristic rules were used also in our case, with switching between them randomly with equal probabilities. Several parameters for the CRS method has to be set before registration and had to be determined experimentally.

- Maximum number of iterations if convergence is not reached: was set to 2000.
- Function convergence tolerance: defined as [29]
  (7) $$|v_{i}-v_{N/2}|\;<\;\epsilon ,$$
  where *v*_*i*_ and *v*_*N*/2_ are the metric values for the first and middle population members of sorted-out population. *N* is the population size.
- Population size: number of members (or “active” solutions); it was set to 100 at the coarse level and 400 at the fine level of registration.
- Population to stop: the number of solutions in the population that satisfy the condition. This parameter was set to *N*/2: half of the population size *N*.

The used registration approach utilizes a multiscale approach, widely used to improve speed, accuracy, and robustness [14]. The basic idea is that registration is first performed at a coarse scale (low-spatial resolution). The spatial mapping determined at the coarse level is then used to initialize registration at the next finer scale. This process is repeated recursively at finer levels until it reaches the finest possible scale. The advantage is that the registration with respect to large-scale features is achieved first and then small corrections are made for finer details. On the other hand, this multiscale strategy fails if a false match is identified on a coarser level [14].

For AF and IR images, we set only one coarse level with decimal subsampling, where only translations *t*_*x*_ and *t*_*y*_ are considered as the optimization parameters. At the next finest scale, all the above-mentioned parameters are considered.

## 4. REGISTRATION RESULTS

The proposed approach with the above parameters was tested on our database of 131 ophthalmologic image pairs (AF and IR images). These images were obtained in low (512 × 512 pixels) or high (1024 × 1024 pixels) resolution mode. High-resolution images were decimated to low resolution, taking every second pixel, to speed up the computation.

**Figure 6:**
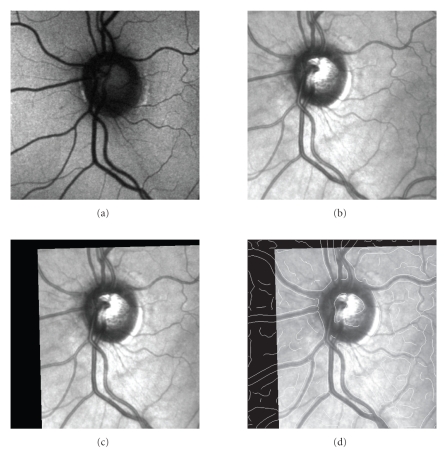
AF-IR registration results: (a) AF image, (b) IR image, (c) registered IR image, (d) edge image for evaluation.

**Figure 7:**
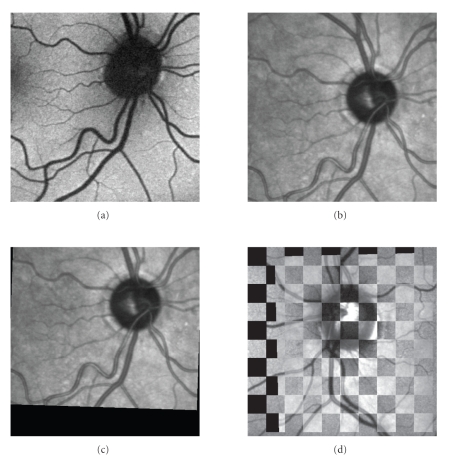
AF-IR registration results: (a) AF image, (b) IR image, (c) registered IR image, (d) mosaic image for evaluation.

The CRS optimization was run 5 times in each case of 30 image pairs (randomly selected from 131 image pairs) to test the sensitivity to random initialization. No visible changes among these particular results were observed. Unfortunately, verification of registration algorithms accuracy is not trivial, because the correct geometrical alignment is not known. Our verification was based on the visual inspection of the edge images and the mosaic images. Examples of registration results are shown in Figures 6 and 7 together with the edge and mosaic images, used for visual evaluation. Each registered pair was classified according to [25] as “poor,” “moderate,” “good,” and “excellent,” or, more precisely, as follows.

- Excellent: the best quality with no visible discrepancy between both images.
- Good: small misalignment between the images in the range of 1 to 5 pixels.
- Moderate: higher misalignment between the images in the range of 6 to 15 pixels.
- Poor: registration with significant misalignment.

The possible pixel misalignments were examined primarily in the area around the optical disc because this area is most important in glaucoma diagnosis. Two experts (A, B) evaluated independently the registration results using the edge and mosaic images simultaneously. The weighted mean scores were determined for each expert. The evaluation results are summarized in Table 1.

## 5. PIXEL LEVEL IMAGE FUSION

Pixel level image fusion means merging the information from several images into a single image. There are many different areas of image fusion, namely, thermal and visual image fusion [30]; remote sensing [31, 32]; medical imaging [33], particularly MRI T1-weighted, T2-weighted, and proton density images [34], SPECT/PET-CT images [35]; and also multimodal retinal images [3, 20].

The aim of the AF-IR image fusion process is to provide a single image with extended information content for the glaucoma diagnosis process. Generally, image fusion is particularly useful for reducing the workload of human operators because it offers different information content in one image. The greatest benefit of the image fusion is achieved if the component images contain complementary information, which applies to AF-IR image pairs. The AF image contains information about the zones with high autofluorescent activity and more visible periphery blood vessels while the IR image carries the information about the optical disc border, its structure, and blood vessels inside OD. The important point is the knowledge of the mutual positions of the autofluorescence zones with respect to optic disc border.

Without fusion, the physician must move his eye between images and it may be difficult to recognize the relationship among patterns and objects, particularly, OD border and AF zones. To prevent this, we tested two pixel-wise fusion processes that are very fast, using artificial color mapping applied on the two mentioned gray-scale component images.

### 5.1. HVS fusion method

**Figure 8:**
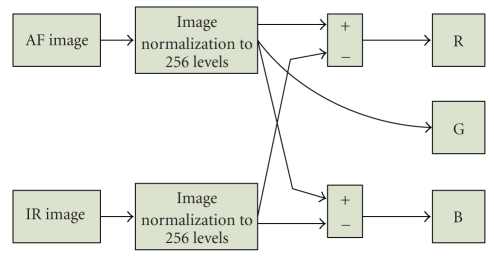
AF-IR fusion scheme.

This approach is similar to the method used in [20], where the fusion was used for color fundus and angiography images. This scheme arises from the biologically motivated pixel-wise fusion methods [33] (HVS stands for the human visual system). For our AF-IR fusion application, the scheme is shown on Figure 8. First, the image normalization to 256 levels is performed. Consecutively, the red channel is computed as a difference between IR and AF images, which enhances the information present in IR image. The blue channel is the negative of the previous combination. The green channel is the AF image itself, because the zones with higher autofluorescency play an important role in early diagnosis of glaucoma and the fusion is thus performed with visually emphasized AF components.

### 5.2. TV-IR fusion method

This scheme is based on a method used for gray-scale video (TV) and infrared (IR) images as in [30]. The common component of the images is computed as the morphological intersection:

(8) f∩g = Min{f(i, j), g(i, j)}.

The characteristic components f* or g* of each image remain after subtraction of the common component:

(9) $$f^{*}\;=\;f-f\cap g,\;\;g^{*}\;=\;g-f\cap g.$$

The characteristic component can be emphasized in the fused image *h* by subtracting each of them from the other image, so that the RGB components are then defined as in [30]:

(10) $$h\;=\;\left(\begin{array}{c} R\\ G\\ B \end{array}\right)=\left(\begin{array}{c} f-g^{*}\\ g-f^{*}\\ 0 \end{array}\right).$$

**Figure 9:**
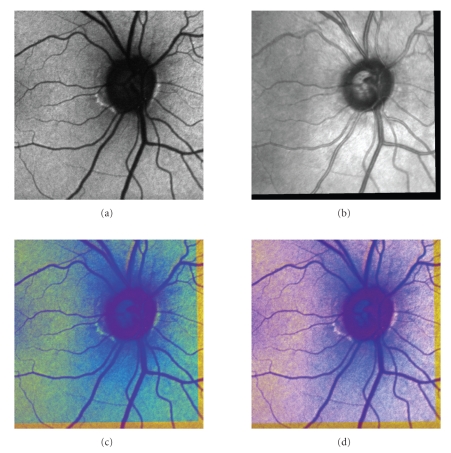
Example of AF-IR fusion results: (a) AF image, (b) IR image, (c) HVS method, (d) TV-IR method.

**Figure 10:**
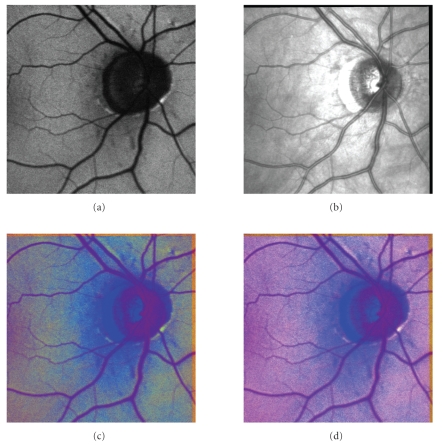
Another example of AF-IR fusion results: (a) AF image, (b) IR image, (c) HVS method, (d) TV-IR method.

We modified this definition in the similar way as in the HVS method to enhance the AF zones in the fused image:

(11) $$h\;=\;\left(\begin{array}{c} R\\ G\\ B \end{array}\right)=\left(\begin{array}{c} f-g^{*}\\ f\\ g-f^{*} \end{array}\right),$$

where *f* represents the AF image, *g* represents the IR channel. For all channels, the normalization to 256 levels is performed before displaying. The example results of the image fusion are shown on Figures 9 and 10, where the original (registered) AF and IR images are shown together with the corresponding fusion results.

## 6. DISCUSSION

To discuss the results of the registration phase, let us concentrate to Table 1. One can see that even considering only the class 1, the percentage of good registration is satisfactory. Classes 2 and 3 include images where the misalignment errors were visible at the periphery of the images and were in the range of several pixels. The application described in previous works [5, 36] uses only the area around the optical disc for analysis of the autofluorescent zones. Therefore, it is important to register precisely the central part (OD with its surroundings). In that sense, class 2 can also be considered well registered and the percentage of successfully registered images is thus 93.1% and 90.8%, respectively (including Classes 1 and 2).

By detailed analysis of images in class 3, we can conclude that the rare wrong registration results arise from blurred IR images, images with small overlap and for images with small but complex distortions, where more generic flexible registration is needed.

Investigating the images with high registration errors in class 4, we can conclude that significant misalignment is found in pairs of images where the IR image is blurred and has a low contrast and the AF image is also blurred or dark. Also, in some cases, a visible strong flexible distortion is present.

As for the preliminary evaluation of the fusion, one can see that the results of the fusion process are very similar for HVS and TV-IR fusions methods. Considering the aim of the fusing (i.e., human analysis of the AF zones with respect to OD border), the TV-IR scheme seems to be more convenient because of higher contrast of AF zones (see Figure 9). This can be observed in most cases of the used database.

## 7. CONCLUSION

A robust framework for registration of AF and IR images was proposed and experimentally verified, including the image preprocessing. The parameters of this approach were optimized for routine use in ophthalmologic clinics. The framework is the part of custom-made software, which is currently used at the Department of Ophthalmology, Friedrich-Alexander University of Erlangen-Nurnberg, Germany. A reasonable trade-off between the speed of computation and registration accuracy was achieved. The computation time for a single registration is about 1 minute at *Intel*, *Pentium M*, 1.7 MHz. The computation time for fusion is negligible. It has been found in clinical trial that the TV-IR-fused images can serve well as a support for the diagnosis and for physician during segmentation process.

## Figures and Tables

**Algorithm 1:**
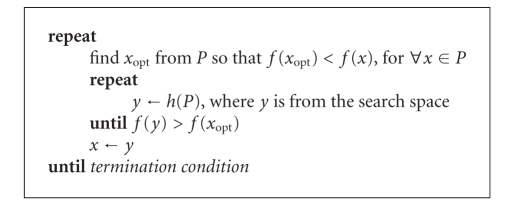
Generate population *P* = *x*_*n*_, that is, *N* random points from the search space *X*.

**Table 1:** Registration results.

Class expert	1	2	3	4	Mean score	Class 1,2,3
A	107 (81.7%)	15 (11.4%)	6 (6.4%)	3 (2.3%)	1.27	128 (97.7%)
B	98 (74.8%)	21 (16.0%)	4 (3.1%)	8 (6.1%)	1.41	123 (93.9%)
